# Predicting needlestick and sharps injuries and determining preventive strategies using a Bayesian network approach in Tehran, Iran

**DOI:** 10.4178/epih.e2018042

**Published:** 2018-08-20

**Authors:** Hamed Akbari, Fakhradin Ghasemi, Hesam Akbari, Amir Adibzadeh

**Affiliations:** Health Research Center, Life Style Institute, Baqiyatallah University of Medical Sciences, Tehran, Iran

**Keywords:** Needlestick injuries, Accident prevention, Bayes theorem, Iran

## Abstract

**OBJECTIVES:**

Recent studies have shown that the rate of needlestick and sharps injuries (NSIs) is unacceptably high in Iranian hospitals. The aim of the present study was to use a systematic approach to predict and reduce these injuries.

**METHODS:**

This cross-sectional study was conducted in 5 hospitals in Tehran, Iran. Eleven variables thought to affect NSIs were categorized based on the Human Factors Analysis and Classification System (HFACS) framework and modeled using a Bayesian network. A self-administered validated questionnaire was used to collect the required data. In total, 343 cases were used to train the model and 50 cases were used to test the model. Model performance was assessed using various indices. Finally, using predictive reasoning, several intervention strategies for reducing NSIs were recommended.

**RESULTS:**

The Bayesian network HFACS model was able to predict 86% of new cases correctly. The analyses showed that safety motivation and fatigue were the most important contributors to NSIs. Supervisors’ attitude toward safety and working hours per week were the most important factors in the unsafe supervision category. Management commitment and staffing were the most important organizational-level factors affecting NSIs. Finally, promising intervention strategies for reducing NSIs were identified and discussed.

**CONCLUSIONS:**

To reduce NSIs, both management commitment and sufficient staffing are necessary. Supervisors should encourage nurses to engage in safe behavior. Excessive working hours result in fatigue and increase the risk of NSIs.

## INTRODUCTION

Needlestick and sharps injuries (NSIs) are known to be one the most important hazards threatening the health and safety of nurses. Transmission of blood-borne infectious diseases, such as hepatitis B, C, and human immunodeficiency virus, is the main concern regarding such injuries [1]. Furthermore, such injuries may also have psychiatric consequences [2] and pose an economic burden [3]. Accordingly, NSIs should be considered seriously and efforts should be made to reduce their occurrence as much as possible.

Like other accidents, NSIs are a multifactorial phenomenon, and many factors and deficiencies at various organizational levels can contribute to such accidents. Job stress, inadequate skills, ignoring the necessary precautions [4], organizational factors [5], work scheduling [6], insufficient staffing [7], guidelines and procedures [8], fatigue [9], poor safety climate [10], and safety training [11] are some factors that have been reported by previous studies to be related to NSIs. Because many factors contribute to NSIs, the rate of such injuries remains high [12], and interventional programs have commonly failed to accomplish their intended objectives.

Currently, organizations tend to use predictive models to manage and reduce injuries and accidents in the workplace [13-15]. Therefore, similar to other accidents and injuries, predictive models can be useful for predicting and preventing NSIs. Several approaches can be used, such as structural equation modeling (SEM), artificial neural network (ANN) modeling, and others. However, in the present study, a Bayesian network (BN) modeling approach was utilized because of its advantages over other modeling approaches (more information about this approach is provided in Supplementary Material 1), including SEM and ANN, which have been emphasized in many studies. SEM is widely used as a modeling approach for testing various hypotheses in social sciences, psychology, and human behavioral studies, but it has several downsides. As illustrated by Zheng & Pavlou [16] and Lee et al. [17], SEM has 3 main limitations, including an inability to infer causal relationships, a restrictive model structure, and the ability to model only linear relationships. All 3 of these limitations are overcome by BN analysis. The representation of causal relationships in the form of conditional probability tables is the bedrock of BN models. Moreover, BNs are very flexible in terms of model structuring. The structure can be determined in various ways, such as using observational datasets, referring to expert opinions, and drawing upon well-known, previously established frameworks, as in the present study. Lastly, in contrast to SEM, relationships in BN models are not reliant on any form of functions, meaning that non-linear relationships can also be modeled. In contrast to ANNs, the causal relationship between each pair of variables can be determined using a BN model, while in an ANN analysis, there is 1 input layer (consisting of input variables or predictors), 1 or more hidden layer, and an output layer (consisting of variables that should be predicted). In other words, in an ANN model, we cannot determine the causal relationships between input variables, and they are considered to be somewhat independent from each other. In addition, some researchers, such as Correa et al. [18], have attempted to compare these 2 approaches. They concluded that the BN model is superior to the ANN model in many aspects, including model performance and the capability to perform various inferences. Besides, an important and unique advantage of BNs over other methods, including SEM and ANN, is its capability for belief updating by introducing new evidence, which is a feature that helps make decisions in contradictory situations. In fact, BN models can also be utilized as decision-making tools, and it has been shown that BN models are more powerful than conventional decision-making approaches such as the analytic hierarchy process and analytic network process [19].

Accordingly, the BN approach would seem to be an ideal option. Using this approach satisfies the following desiderata: first, it is obvious that many factors from various organizational levels are able to contribute to NSIs, and using the BN modeling approach, all these variables can be included in the model; second, the causal relationships between each pair of variables can be specified; lastly, in contrast to other approaches, preventive strategies for reducing the risk of NSIs can be explored [20,21].

Accordingly, the aim of the present study was to build a BN model based on the most important factors for predicting NSIs and also to find strategies for reducing the rate of such injuries.

## MATERIALS AND METHODS

This cross-sectional study was conducted in 5 hospitals located in Tehran, Iran. Participation was voluntary and the convenience sampling method was used to select participants. All hospitals were governmental and located in different regions of Tehran. Data were collected during summer 2017. All participants signed an informed consent form. Moreover, the study was supervised by the ethical committee of Baqiyatallah University of Medical Sciences (ethical code: IR.BMSU.REC.1395.378).

In the first step, we reviewed previous studies, and based on them, selected some factors that can affect the occurrence of NSIs. The following variables were selected: management commitment to safety (the relative importance of safety at higher levels of the organization), staffing (the adequacy of staff to carry out the needed tasks within an organization), the availability of safe work procedures for disposing needles or safety devices, night shifts per month and working hours per week (representing the quality of work scheduling), fatigue, supervisors’ attitude toward safety (how supervisors deal with safety issues in the hospital), environmental conditions (cleanness, housekeeping, and so on), safety training, teamwork (collaboration among nurses), and safety motivation. Moreover, only NSIs that had occurred within 3 months before the study were considered.

In the second step, a self-administered questionnaire was validated for measuring the variables. The questionnaire was validated using indices such as the content validity ratio and content validity index [22,23]. The opinions of 6 experts, including 3 occupational health and safety practitioners experienced in occupational health and safety issues in hospitals and 3 university professors, were elicited in this step. The final questionnaire contained 5 items for measuring management commitment to safety, and 4 items used for measuring each of the other variables. These questions were scored using a 5-point Likert scale. The average of the items’ scores was used as the final score of each variable, as has been done in several previous studies [24,25]. The availability of procedures, number of night shifts per month, working hours per week, and whether a participant had experienced an NSI in the 3 months before the study were measured directly by asking a single question for each variable. Furthermore, the Cronbach alpha was used to assess the reliability of the questionnaire. Some of the items used in the questionnaire are presented in Table 1. All variables had acceptable reliability. The Cronbach alpha values for the variables were as follows: 0.88 for management commitment to safety, 0.83 for staffing, 0.82 for safety training, 0.81 for supervisors’ attitude to safety, 0.83 for fatigue, 0.78 for teamwork, 0.76 for safety motivation, and 0.81 for environmental conditions.

**Figure 1.**
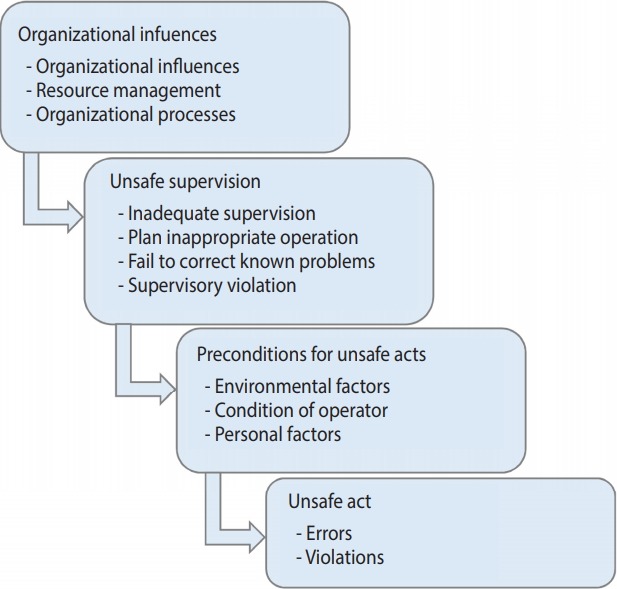
The Human Factors Analysis and Classification System framework.

In the third step, the BN model was constructed. BN is a directed acyclic graph used to represent causal relationships among a set of variables. It has a qualitative and a quantitative part. The qualitative part is composed of nodes and arcs that represent the variables and their causal relationships. The quantitative part contains conditional probability tables, which explain how each variable is affected by its parents [20,26]. For constructing the qualitative part of the BN model (i.e., causal relationships among the variables), the Human Factors Analysis and Classification System (HFACS) framework was used. HFACS is a conceptual framework for assessing accidents occurring in different organizations that aims at providing deep insights into the causation of accidents. According to this framework, any accident occurs because of human errors at various levels of an organization. The framework is composed of 4 levels, with each level is directly affected by the immediately higher level. According to this model, unsafe acts are committed when there are certain preconditions for unsafe acts. Unsafe supervision, in turn, creates such preconditions, and organizational deficiencies result in unsafe supervision [27]. The HFACS framework is presented in Figure 1. Moreover, 2 assumptions are used in this step: first, that variables in each level are independent and have no direct effect on each other; and second, that all variables located in the higher level directly affect all variables located in the immediately lower level. The mapping of the variables onto the HFACS framework is illustrated in Figure 1.

**Figure 2.**
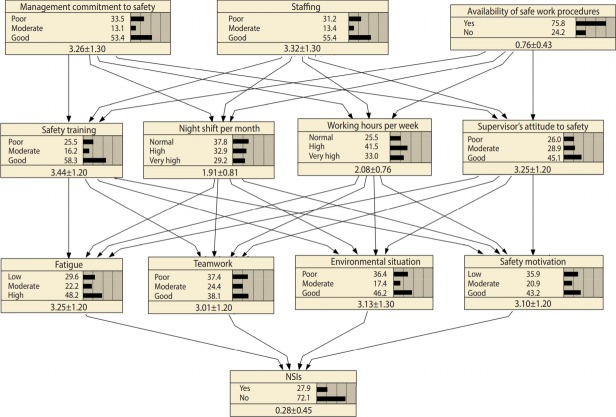
The Bayesian network model for analysis and predicting needlestick and sharps injuries (NSIs) after training the model (the values in the last row of each node represent mean±standard deviation).

In the fourth step, the model was trained based on the dataset provided by the completed questionnaires. Before training the BN model, the continuous variables in the model should be discretized. In the present study, the values of variables measured by the questionnaire could range from 1 (the lowest possible value) to 5 (the highest possible value). This range was divided into 3 equal intervals for each variable. Further information on discretizing the variables is presented in Table 1. The least number of cases needed for training the model depicted in Figure 2 is equal to 3^5^ = 243. The expectation–maximization algorithm [28] was used to train the model. This algorithm can also deal with incomplete datasets, reducing the number of unqualified cases.

Once the structure of the model was determined, the number of required cases could be calculated. The number of initially distributed questionnaires was calculated based on the structure of the BN model (as mentioned above, 243 cases were needed to train the model and at least 50 cases to test its performance), and a conservative prediction was made that 50% of nurses would not respond the questionnaire. Accordingly, 586 questionnaires were distributed among nurses. The number of questionnaires distributed in each hospital was proportional to the size of the hospital.

Once the model was trained, its performance was assessed. The performance of a model refers to its ability to predict new cases. A weak model would not have a favorable performance and could not be generalized for use in other hospitals. In the present study, a set of data that was not used to train the model was used to assess the performance of the model. The error rate, logarithmic loss, quadratic loss, and spherical payoff (SP) were indices used to assess the robustness of the model [29,30]. The error rate is calculated based on the false positive and false negative results of the model. Then, these results are merged and represented in the shape of a table, known as a confusion matrix [30]. SP is calculated using the following equation [30]:

$$\mathrm{SP}\;=\;\mathrm{MOAC}\;\frac{P_{c}}{\sqrt{\sum_{j=1}^{n}{P_{j}}^{2}}}$$

Where, MOAC represents the mean probability value of a given state averaged over all cases, P_c_ represents the predicted probability of the correct state, P_j_ represents the predicted probability of state j, and n is the total number of states. The index ranges from 0 to 1, and higher values are indicative of better model performance. Logarithmic loss is calculated based on the probability of true positive results. This index can range from 0 to infinity, and the lower the value, the better the model performance. SP can range from 0 to 2, and values close to 0 are indicative of better model performance [30,31].

In the next step, a sensitivity analysis was performed to determine how sensitive the output variable was to changes in each of its predictors. Based on this analysis, predictors could be ranked based on their ability to predict the output variable. Predictive reasoning and mutual information were the 2 approaches we used in this step. Mutual information refers to the amount of information shared between 2 variables, and it determines the level of reduction in the uncertainty associated with one variable by gaining knowledge regarding another variable [32].

Finally, some intervention strategies were proposed. Intervention strategies can be simple, focusing on a single variable, or complex or “joint,” attempting to improve several variables simultaneously to improve the output variable. In the present study, based on predictive reasoning, we recommend some intervention strategies to reduce the rate of NSIs. It should be mentioned that belief updating by introducing new evidence is a unique characteristic of BNs that allows them to be used to perform various reasoning processes. Belief updating is based on the well-known Bayes theorem [20]:

pAB=pBA.pAPB

Using predictive reasoning, we can assess the effect of changes in various variables on the output variable. This type of reasoning is normally used for selecting various intervention strategies with the aim of improving the output variable, as in many previous studies [13,24,29]. In the present study, the Netica version 6.05 (https://www.norsys.com/index.html) developed by Norsys was used to perform all calculations and inferences.

## RESULTS

A total of 408 completed questionnaires were received from the hospitals (response rate, 70.0%). Fourteen questionnaires were discarded because of systematic response patterns (i.e., questionnaires where participants seemed to have responded using obvious patterns without reading the questions). This pretreatment of data has been recommended by Seo [33]. Of the remaining questionnaires, 343 were used to train the model, and model performance and its generalization ability were evaluated using the other 50 questionnaires. The nurses who participated in the study were between 23 and 51 years old. They had at least 3 months of experience. Most of them (76.5%) were female, and 30.0% of them had experienced at least 1 NSI during the 3 months before the study.

By mapping the selected variables into the HFACS and based on the 2 assumptions of the HFACS, the network represented in Figure 2 was obtained.

Figure 2 represents the model that we constructed and trained to analyze NSIs. The marginal probabilities for various variables in this figure explain the present circumstances in the hospitals. According to this BN model, 27.9% of nurses had experienced at least 1 NSI in the 3 months before the study, 55.4% felt that the level of staffing in their hospitals was sufficient, and 75.8% of respondents reported that safe work procedures were easily available. Moreover, 43.2% of participants were motivated to be involved in safety-related activities.

After training the model, it is important to assess its performance (i.e., its ability to predict new cases). As explained above, a dataset containing 50 cases was used to test the model. The performance of the model based on various indices is presented in Table 2. It should be stressed that no cut points have been recommended for rejecting or accepting a BN model based on these indices, but when we compare them with those reported by other studies, such as those conducted by Mohammadfam et al. [29] and Dlamini [31], it can be concluded that the model showed an acceptable performance.

In the next step, a sensitivity analysis was performed to determine which variables had stronger effects on NSIs. The results of the sensitivity analysis based on both predictive reasoning and mutual information are presented in Table 3. Motivation to participate in safety-related activities, fatigue, and physical environment had a larger effect on NSIs than teamwork. Among the unsafe supervision variables, the effects of working hours per week and supervisors’ attitude to safety were larger than others. Finally, management commitment to safety and staffing both had a strong effect on NSIs, while the effect of availability of safe work procedures was negligible.

As discussed above, we also conducted predictive reasoning to determine which intervention strategies would be more effective in reducing the rate of NSIs. Simple strategies to reduce NSIs are presented in Table 3 (note that the results are for “NSIs= no”). Accordingly, by improving the state of safety motivation to its best state, the rate of NSIs could be decreased to 20.5%. Through the same process, it can be inferred that reducing fatigue would result in a reduction of NSIs to 21.4%. Therefore, the best simple strategy is to focus on improving the motivation of nurses to participate in safety-related activities and to use safety equipment for disposing needles and other sharps, because that was the variable with the strongest influence in the first layer of the BN model and its improvement resulted in the greatest reduction in NSIs. Alongside safety motivation, fatigue was also identified as a variable with a considerable effect on NSIs.

**Figure 3.**
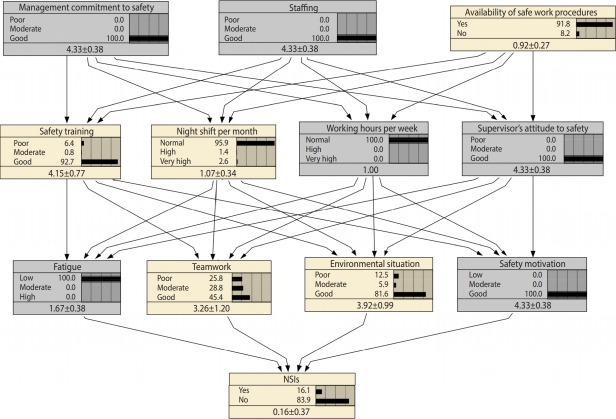
The joint strategy for reducing needlestick and sharps injuries (NSIs) in hospitals (the values in the last row of each node represent mean±standard deviation).

The next question to be answered is how we can motivate nurses to engage in the aforementioned activities or to reduce their fatigue. To answer this question, another sensitivity analysis was conducted on all nodes that directly affected safety motivation. Accordingly, safety motivation was more sensitive to working hours per week and supervisors’ attitude than to the other 2 variables, and fatigue was more sensitive to working hours per week and night shifts per month. Overall, it can be concluded from this set of analyses that supervisors’ attitude and working hours per week were more important than the other 2 variables. Therefore, in order to reduce NSIs, these 2 variables should be improved simultaneously. Using the same procedure, we determined that staffing and management commitment to safety were far more important than the availability of safe work procedures. Therefore, to reducing NSIs, the joint strategy depicted in Figure 3 is recommended.

## DISCUSSION

Many organizational and workplace-related factors can affect NSIs, directly or indirectly. In this study, we used a BN approach alongside the HFACS framework to model and analyze NSIs, as well as to recommend several intervention strategies. The error rate of the model in predicting new cases was 14.00%, which was completely acceptable based on the findings reported by other studies. The results of this study showed that a lack of motivation to participate in safety-related activities and an absence of use of safety devices for disposing needles were the most important causes that immediately affected NSIs. This finding is in line with the results of Cho et al. [34], who reported that the rate of NSIs was significantly lower among nurses who routinely used safety containers. A lack of safety motivation can also be due to several reasons. By performing a sensitivity analysis on the safety motivation node in the present BN, we determined that supervisors’ attitudes toward safety and working hours per week were the 2 variables with the largest effects on safety motivation. Accordingly, in order to promote safety motivation among nurses, supervisors’ attitudes toward safety and working hours per week should be improved. Both these factors are primarily affected by management commitment to safety and the level of staffing. Many previous studies have found relationships between NSIs and organizational factors [35]. Moreover, Smith et al. [36] reported that the hospital safety climate was associated with NSIs. Since management commitment to safety is an integral part of hospital safety climate and probably its most important dimension, the results of the present study are consistent with those reported by Smith et al. [36]. There are various ways through which hospital managers can show their commitment to safety. Writing a policy statement regarding the commitment of management to nurses’ occupational health and safety may be the first step. Some other possibilities include providing high-quality safety training; investigating every NSI without blaming the employees, but with the goal of preventing it from reoccurring; and assigning incentive systems to reward employees who propose practical recommendations for improving hospital safety. In fact, supervisors’ attitudes toward safety are a good sign of management commitment and is the element of the chain that links managers’ attitudes to employees’ behavior, so strong management commitment to safety should be reflected in the attitudes and behavior of supervisors, which motivate and encourage nurses to engage in safety behavior. In addition to supervisors’ attitudes, various tactics can be implemented to motivate employees to participate in safety-related activities, to use safety devices for disposing needles, to avoid recapping, and to report NSIs. Using new and creative incentive systems can be effective in this regard [37]. Increasing knowledge through safety training is another promising technique, as many studies have emphasized the role of knowledge in improving behavior. However, in the present study, we found that safety training had less effect on motivation than other variables. This finding demonstrates that these training courses suffer from a lack of quality, so the content of these training courses should be modified.

According to the present study, fatigue had a considerable effect on NSIs, and was mainly influenced by working hours per week and night shifts per month. The results are in line with those reported by the study carried out by Lo et al. [38], in which a significant relationship was found between long work hours, chronic insomnia, and NSIs. Accordingly, unsupervised and unsafe high working hours can cause fatigue, disturb nurses’ sleep, and eventually increase the risk of NSIs. Therefore, to reduce fatigue, it is necessary to control nurses’ working schedule.

As in most previous studies, the present study has some limitations that should be addressed in future studies. The present study was conducted in 5 hospitals, but it would be better to include more hospitals in the study. Because we assessed some managerial and situational factors, including more hospitals would result in more diverse data, which would demonstrate the effects of the variables more precisely. There are some other variables that can affect NSIs, but were not included in the present model. Leadership and incentive systems are some of these variables, the effects of which can be assessed in future studies. Moreover, the same model could be constructed for assessing nursing errors and variables influencing those errors, which is a promising area for future research.

In conclusion, to reduce NSIs, both management commitment and sufficient staffing are necessary. Supervisors should encourage nurses to engage in safe behavior. Excessive working hours result in fatigue and increase the risk of NSIs.

## Figures and Tables

**Table 1.** Mapping the variables to the HFACS framework

Variables	Selected items	Location in HFACS	States
Management commitment to safety	Management provides all necessary measures to prevent NSIs Management always emphasizes that all NSIs should be reported	Organizational climate (level 4)	Poor, moderate, good
Staffing	The amount of personnel in our unit is enough to perform the needed tasks	Resource management (level 4)	Poor, moderate, good
	I am responsible for many tasks, so I should always work fast		
Availability of safe work procedures	Safe work procedures for disposing used needles or using safety devices are always readily available in our hospital	Organizational processes (level 4)	Yes, no
Night shifts per month		Plan inappropriate operation (level 3)	Normal (≤8), high (9-11), very high (≥12)
Working (hr/wk)		Plan inappropriate operation (level 3)	Normal (<45), high (45-55), very high (>55)
Supervisors’ attitude toward safety	My supervisor always follows safe work practices	Supervisory violation (level 3)	Poor, moderate, good
	My supervisor always talks to me about the benefits of using safety devices in disposing needles and manipulating sharp equipment		
Safety training	Pre-employment training courses have informed us about the importance of NSIs	Inadequate supervision (level 3)	Poor, moderate, good
	I received high-quality training regarding how NSIs can be pre- vented		
Fatigue	I always feel tired at work	Condition of operators (level 2)	Low, moderate, high
	I do not have enough time to fully rest		
Teamwork	There is good collaboration between me and my colleagues	Personal factors (level 2)	Poor, moderate, good
	My colleagues always inform me about how to prevent NSIs		
Physical environment	My working environment is noisy and distracting	Environmental factors (level 2)	Poor, moderate, good
	My working environment suffers from poor housekeeping		
Safety motivation	I always prefer to use a safety box or other safety devices for disposing needles	Condition of operators (level 2)	Poor, moderate, good
	I like to participate in activities that can improve my occupational health and safety and prevent NSIs		
NSIs	Have you experienced an NSI in the last 3 months?		Yes, no

HFACS, Human Factors Analysis and Classification System; NSI, needlestick and sharps injury.

**Table 2.** Performance of the model

Index	Value	Range (acceptance criteria)
Total error rate (%)	14.00	0-100 (the lower the better)
Logarithmic loss	0.35	0-infinity (the lower the better)
Quadratic loss	0.23	0-2 (the lower the better)
Spherical payoff	0.87	0-1 (the higher the better)

**Table 3.** Sensitivity analysis based on mutual information and predicitive resoning

Variables	The posterior probability of “NSIs = no”		Sensitivity	Mutual information (%)
	Worst state	Best state		
Preconditions for unsafe acts				
Safety motivation	62.8	79.5	26.6	0.019 (2.3)
Fatigue	65.3	78.6	20.4	0.016 (1.8)
Teamwork	67.2	75.3	12.1	0.005 (0.6)
Physical environment	66.7	79.4	19.0	0.017 (2.0)
Unsafe supervision				
Supervisors’ attitude to safety	59.6	77.7	30.4	0.019 (2.3)
Safety training	66.5	74.8	12.5	0.004 (0.5)
Working hours per week	63.8	78.8	23.5	0.013 (1.5)
Night shifts per week	64.9	75.7	16.6	0.007 (0.9)
Organizational influences				
Staffing	65.2	76.0	16.6	0.008 (1.0)
Management commitment	66.0	75.8	14.8	0.007 (0.8)
Availability of safety procedures	71.9	72.2	0.4	0.000 (0.0)

NSIs, needlestick and sharps injuries.

## SUPPLEMENTARY MATERIALS

Supplementary Material 1: BN modeling approach is available at http://www.e-epih.org/.
